# Supplementary material for: A Critical View of the Application of the APEX Software (Aqueous Photochemistry of Environmentally-Occurring Xenobiotics) to Predict Photoreaction Kinetics in Surface Freshwaters
Source: Molecules. 2019 Dec 18;25(1):9. doi: 10.3390/molecules25010009 (PMC7017383; doi:10.3390/molecules25010009)
Supplement: Supplementary file 1 [file molecules-25-00009-s001.zip › 3.2.4_gcc-4.4.0.zip!/3.2.4_gcc-4.4.0/3.2.4_gcc-4.4.0/doc/octave/html/interpreter/Rational-Approximations.html]

Rational Approximations - Untitled


Previous: Simple File I/O,
Up: Basic Input and Output


---

#### 14.1.4 Rational Approximations

— Function File: s = **rat** (x, tol)  
— Function File: [n, d] = **rat** (x, tol)  
> Find a rational approximation to x within the tolerance defined
> by tol using a continued fraction expansion. For example,
>
> ```
>           rat(pi) = 3 + 1/(7 + 1/16) = 355/113
>           rat(e) = 3 + 1/(-4 + 1/(2 + 1/(5 + 1/(-2 + 1/(-7)))))
>                  = 1457/536
> ```
>
> Called with two arguments returns the numerator and denominator separately
> as two matrices.


**See also:** rats.

— Built-in Function: **rats** (x, len)  
> Convert x into a rational approximation represented as a string.
> You can convert the string back into a matrix as follows:
>
> ```
>              r = rats(hilb(4));
>              x = str2num(r)
> ```
>
> The optional second argument defines the maximum length of the string
> representing the elements of x. By default len is 9.
>
>
>
> **See also:** format, rat.
